# Humans versus machines: Who is perceived to decide fairer? Experimental evidence on attitudes toward automated decision-making

**DOI:** 10.1016/j.patter.2022.100591

**Published:** 2022-09-29

**Authors:** Christoph Kern, Frederic Gerdon, Ruben L. Bach, Florian Keusch, Frauke Kreuter

**Affiliations:** 1 School of Social Sciences, University of Mannheim, A5, 6, 68159 Mannheim, Germany; 2 Mannheim Centre for European Social Research (MZES), University of Mannheim, A5, 6, 68159 Mannheim, Germany; 3 Department of Statistics, Ludwig-Maximilians-Universität München, Ludwigstrasse 33, 80539 Munich, Germany; 4 Joint Program in Survey Methodology, University of Maryland, 1218 LeFrak Hall, 7251 Preinkert Drive, College Park, MD 20742, USA

**Keywords:** automated decision-making, fairness perceptions, experiment, survey, fair machine learning, algorithms

## Abstract

Human perceptions of fairness in (semi-)automated decision-making (ADM) constitute a crucial building block toward developing human-centered ADM solutions. However, measuring fairness perceptions is challenging because various context and design characteristics of ADM systems need to be disentangled. Particularly, ADM applications need to use the right degree of automation and granularity of data input to achieve efficiency and public acceptance. We present results from a large-scale vignette experiment that assessed fairness perceptions and the acceptability of ADM systems. The experiment varied context and design dimensions, with an emphasis on who makes the final decision. We show that automated recommendations in combination with a final human decider are perceived as fair as decisions made by a dominant human decider and as fairer than decisions made only by an algorithm. Our results shed light on the context dependence of fairness assessments and show that semi-automation of decision-making processes is often desirable.

## Highlights

• Investigates public acceptance of automated decision-making across four contexts
• Experimentally varies degree of automation, used data, and decision type and context
• Uses experimental data from a probability-based sample with large sample size
• Finds that respondents overall prefer human involvement

## The bigger picture

Public institutions and businesses increasingly rely on algorithmic support to make decisions about citizens. Promising enhanced efficiency, organizations use automated decision-making (ADM) for purposes ranging from content recommendations to credit or bail decisions. However, algorithms may potentially worsen social inequities by reproducing biases they find in the data. Moreover, citizens may feel uncomfortable being judged by a “machine,” but they also may distrust, e.g., the objectivity of human deciders. We present a survey experiment on citizens’ preferences for the degree of involvement of algorithms and human deciders across four highly relevant ADM contexts, while varying two other situational parameters. We find that respondents prefer the involvement of a human decider to purely automated decisions. Depending on context, ADM designers should therefore consider involving a human decider in the process.

## Graphical abstract

**Figure:**
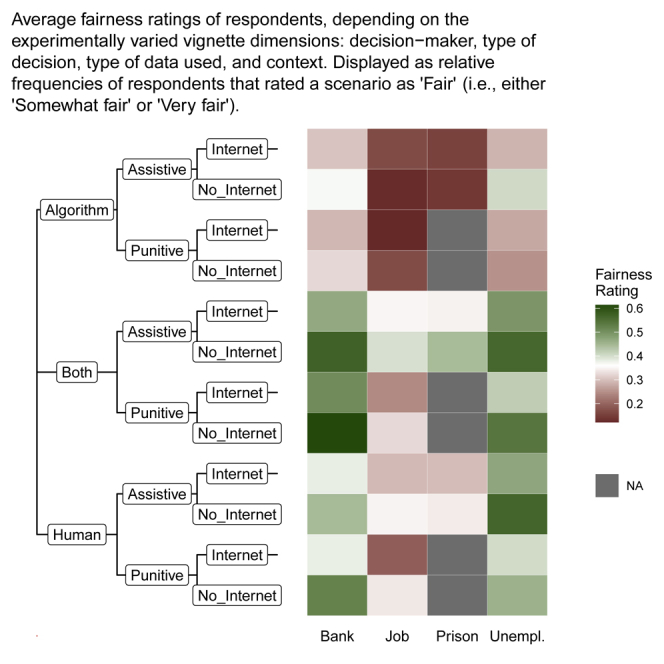

## Teaser

Organizations increasingly use algorithms to make decisions on individuals, e.g., for credit or bail decisions. These automated decision-making (ADM) systems need to be fair and publicly accepted to be successful in the long run. We experimentally investigate several factors that may affect public acceptance of ADM applications across four highly relevant contexts. We find an overall preference for decisions that involve a human decider compared with purely automated decisions.

## Introduction

Automated decision-making (ADM) is increasingly used in many critical domains that affect individuals’ life chances. This includes the use of machine learning (ML) to support public employment services,^1^ algorithmic decision-making in human resources (HR) management,^2^ and (infamous) examples of automated risk assessments in criminal sentencing.^3^ Against this backdrop, research on fairness in ML has recognized that fairness of ADM systems needs to be evaluated within the social contexts in which they are placed.^4^ The successful implementation of ADM in a given setting requires public support and support of the affected individuals. Beyond risk assessments,^5^ fairness and acceptability evaluations critically guide discussions on whether and how ADM solutions should be employed in a given context.^6^ Likewise, fairness perceptions inform developers in designing socially accepted ADM systems and policy-makers in considerations on which application contexts are deemed sensitive and need particular (legal) attention.

Multiple design features of the ADM system may affect acceptance. ADM outputs may constitute the final decision or may be used as a recommendation for an action. In other instances, computer programs may simply provide data without suggesting a recommendation or classification. It is likely that context and other characteristics of the concrete ADM system influence whether people deem it acceptable if an ADM actually decides on its own or to which extent human supervision and intervention are desired. People are also likely to vary in their perceptions of the ADM system depending on their own experiences, understanding, and likelihood of being affected by these systems. People from groups who have been discriminated against in the past may particularly worry about unfair or otherwise biased decisions.

Previous research has examined fairness perceptions with respect to selected application contexts, fairness metrics, and explanation styles (see background and related work). The study presented here aims to connect the different findings and lines of previous research. Our focus is on perceptions toward the system as a whole, i.e., whether ADM is perceived to be fair and acceptable to be applied for a specific purpose and in a specific context. Novel is the measurement of fairness assessments in a survey experiment that considers three degrees of human involvement in decision-making across several application contexts, while varying further design features within each context. This set-up allows the examination of interactions between application contexts and characteristics of the ADM approach. Novel is also the combined analysis of fairness ratings in interaction with characteristics of the evaluating individuals, where individuals are drawn from the population at random with known selection probabilities, improving the external validity of our findings.

More specifically, we compare perceptions and acceptance of the use of ADM systems across four different contexts (banking, HR, criminal justice, and employment agencies). We experimentally research scarcely investigated differences in acceptance between mainly human decision-making, semi-ADM, and fully ADM. We furthermore elucidate whether assistive decisions are deemed fairer than punitive decisions, and we explore inter-individual heterogeneity in responses. The main questions we answer are: first, which degree of automation is more accepted/perceived fairer across scenarios and situations? Second, do individual characteristics interact with context and design characteristics in affecting acceptance/perceived fairness?

We find that semi-ADM is perceived as fairer than fully ADM and roughly as fair as mainly human decision-making. In addition, the preference for human oversight varies by context. These results not only suggest that ADM systems need to be evaluated on a case-by-case basis, but they also provide directions for initial design choices that increase the chance of public acceptance according to specific design categories of interest. In summary, we provide the following contributions to research on public perceptions toward ADM:

• Comparison of perceptions toward different levels of automation in decision-making processes across contexts, providing implications for how to design ADM applications depending on context
• Insights into acceptance of assistive and punitive types of decisions across contexts, showing in which cases human involvement should be particularly considered in ADM design
• Data based on an experimental approach within a nationally representative probability-based sample with known selection probabilities and a larger sample size than (most) previous research, thus providing a high-quality sample

### Background and related work

Research on fairness in ML and ADM focused so far primarily on important technical aspects of fairness, such as defining and choosing fairness metrics, evaluating existing ADM applications with respect to their fairness implications, and correcting unfair systems (see, e.g., Barocas et al.^7^ for an overview on fair ML). Other studies have investigated the legal preconditions of using algorithmic systems,^8^ provided philosophical perspectives on fairness in algorithmic decision-making,^7^^,^^9^ or investigated trust in algorithmic systems in human-machine interactions.^10^ However, over the past years, a strand of literature has emerged that investigates human perceptions on fairness in ADM, i.e., how individuals from the populations potentially affected by ADM systems evaluate their use.

A literature review by Starke et al.^11^ identified several papers that investigated humans’ perceptions of algorithmic fairness. We focus on four key dimensions that have been investigated with respect to perceptions of algorithmic fairness: (1) the context in which an ADM system is applied and the type of impact the system makes, (2) the degree of human involvement in decision-making, (3) the features used by an algorithm, and (4) the characteristics of the individual that may influence perceptions of algorithmic fairness.

The first dimension is concerned with the contexts in which ADM systems are used and the impact of a decision for an individual’s life.^11^^,^^12^ Previous research highlighted that empirical results on perceptions in specific ADM contexts may not translate into other contexts, cautioning researchers against over-generalizations.^10^ Although each context comes with myriads of idiosyncrasies, it appears likely that the stakes of the decision-making context are one crucial differentiating factor. In an exploratory study, Smith et al.^13^ found that fairness of ADM systems matters less to individuals when the decisions to be made have relatively little impact, such as in music and movie recommendations, while fairness plays a much larger role when the decisions have relatively large impact, such as in job recommendations. Likewise, recent advances in fair ML emphasize that specific types of prediction error may matter more for some kinds of decisions than for others: for assistive actions, avoiding false negatives might be viewed as critical; for punitive actions, avoiding false positives might be considered most important.^14^^,^^15^ Translating this notion into fairness perceptions by drawing on insights from economics, individuals may attribute higher weight to potential losses following from decisions than to potential gains.^16^

Relating to the second dimension, some research exists on direct comparisons between human and (purely) ADM for specific contexts^11^ and concludes that there is great variation in relative perceived fairness across contexts and that characteristics of the task impact fairness perceptions. In a series of survey experiments, Nagtegaal^17^ found that public sector employees perceived human decision-makers as procedurally fairer for tasks with high complexity, and that adding an algorithm to a human in the decision-making process may increase justice perceptions. In another experiment, participants deemed human decisions as fairer than algorithmic decisions with tasks that particularly required human skills (hiring and work evaluations), while no difference was found for perceived fairness relating to “mechanical” skills (work assignment and scheduling).^18^ Research that compares hybrid decision-making (which involves both algorithmic and human decision-making) with solely algorithmic or human decision-making across contexts is scarcer. For instance, Gonzalez et al.^19^ find that combined decision-making is preferred over completely ADM in hiring decisions, but this also depends on the familiarity of the respondent with artificial intelligence (AI). Similarly, another study in the HR context finds that individuals have negative attitudes to purely ADM because of the limited use of information by ADM systems.^20^ With an Amazon MTurk sample, Starke et al.^11^ found that ADM decisions overseen by a “privacy professional” increased perceived legitimacy of the decision compared with purely algorithmic or human decisions. Overall, a literature review by Langer and Landers^21^ suggests that hybrid decision-making is preferred over fully ADM, at least in specific contexts. However, the review study by Starke et al.^11^ finds no clear public preference for whether solely human decision-making or a hybrid process involving humans and algorithms was preferred and conclude that no general statement on the preference for either human or ADM could be made. The literature may therefore profit from a systematic comparison of degrees of automation in several major ADM applications contexts with a large and probability-based sample.

The third dimension is concerned with which features, i.e., which variables and therefore also individual characteristics, an algorithm draws on. Dodge et al.,^22^ for example, find in a qualitative study that, among others, the appropriateness of the data basis and the features used and not used by the algorithm matter to people’s fairness perceptions. Grgic-Hlaca et al.^23^ suggest, based on their reading of the literature, eight feature properties (e.g., reliability and privacy sensitivity) that may be relevant for fairness perception. Using a survey, the study also finds that most of these properties matter for fairness perceptions, and survey respondents agreed that the use of reliable, relevant, or private information was fair. Furthermore, previous studies have shown that the fairness of data use depends on the proximity of the type of data to the system’s purpose in the context of crime,^24^^,^^25^ and that the legitimacy of ADM is higher when purpose-specific rather than general data in the form of individual online browsing behavior are used,^26^ supporting the idea that the normative appropriateness of using personal data is context dependent.^27^

The fourth dimension focuses on the often-neglected perspective of evaluating individuals and their characteristics and experiences. Particularly, the perceived fairness of the use of specific individual characteristics in an ADM application for bail decisions has been shown to correlate with the characteristics of the evaluating individual. For example, women deemed it less fair for the ADM to rely on gender in this case.^28^ Similarly, women are less likely to accept automated university course recommendations that use gender when the results disadvantage women for science course recommendations.^29^ However, a review found no conclusive evidence for general direct effects of gender on fairness perceptions.^11^

Beyond protected attributes, inter-individual differences in perceptions may arise from differing attitudes and knowledge. For instance, higher general privacy concerns may lower the acceptance of data regarded irrelevant for decision-making. Additionally, knowledge about algorithms may increase positive evaluations of the employment of algorithms in decision-making processes.^30^

Our research aims at connecting the different dimensions and lines of previous research by investigating them within a single framework, thereby enabling us to draw conclusions that may hold beyond a single context. In addition, we advance the literature by focusing on the perceived fairness of three degrees of human involvement in decision-making across several contexts with an experimental approach. We compare several application contexts for decision-making between each other, while also investigating preferences within contexts. Because perceptions may strongly differ between contexts, any variation caused by specific characteristics within contexts does not necessarily imply that this specific characteristic will matter for all contexts. Furthermore, we analyze fairness ratings in interaction with characteristics of the evaluating individual. Moreover, in addition to fairness perceptions, we measure acceptance ratings of ADM use cases. We compare responses to both questions, which allows us to learn whether they measure a common latent construct or whether respondents clearly differentiate between fairness perceptions and overall acceptance.

### Data

To investigate the impact of specific characteristics of computationally supported decision-making on people’s acceptance and perceived fairness, we conducted a factorial survey experiment, or “vignette” experiment,^31^ in July 2021 (Wave 54) using the German Internet Panel (GIP), a probability-based longitudinal online survey.^32^ GIP covers both the online and the offline population living in private households in Germany aged 16–75 years, and participants were recruited face-to-face (in 2012 and 2014) and via postal mail (in 2018). People without a computer and/or no access to the Internet in the first two recruitment waves were provided with a basic laptop/tablet computer to participate. Panel members are invited on a bimonthly basis to participate in web surveys on political and economic attitudes and reform preferences.^32^ The Wave 54 questionnaire of the GIP included a rider with our vignette experiment that was specifically developed for this study. A total of 4,108 GIP panel members participated in the Wave 54 survey with a completion rate for GIP Wave 54 of 65.8% (COMR; see American Association for Public Opinion Research^33^). Excluding participants who broke off the survey or did not provide answers to our vignette questions leaves us with 3,930 respondents with valid fairness assessments and 3,972 respondents with complete acceptance ratings.

Being a probability-based survey, the GIP is based on random sampling from a sampling frame from the target population with known selection and known inclusion probabilities.^32^ Several studies found that, in general, probability-based online panels outperform non-probability samples, which are commonly used in research on ADM fairness perceptions, such as Amazon MTurk, in terms of data quality.^34^ As such, the sample of the GIP is a very good representation of the general population in Germany.^35^^,^^36^ Our study design is thus strong in both internal validity, because of the experimental design, and the representativity of the sample, that is, in external validity.

### Vignette experiment

In the vignette experiment, respondents are presented with 4 of 42 text descriptions of hypothetical scenarios on decision-making that suggest different degrees of automation, among others (see below). The descriptions vary by characteristics (or dimensions) that can take on different specified levels; by randomly assigning vignettes to respondents, researchers may estimate the causal effects of changes in single-vignette dimensions on responses.^31^ We created 42 descriptions that were blocked into four groups that each refer to one specific context of ADM applications (representing the dimension *context*). We investigate four contexts that we chose because they have been extensively discussed in academic literature on ADM and, partly, in public discourse, and therefore are of particular relevance. These contexts vary by the potential severity of decisions, i.e., how strongly they may affect citizens’ lives: (1) “Bank,” bank credits and products;^37^, ^38^, ^39^ (2) “Job,” HR decision-making;^2^ (3) “Prison,” criminal justice;^3^^,^^40^^,^^41^ and (4) “Unemployment,” actions of employment agencies.^42^

Each respondent received one randomly drawn vignette for each context in random order. The vignettes further contained the following dimensions: *action*, *data*, and *decision-maker*. Although we argued that an important difference between contexts is the severity of the decision, previous literature points to the importance of whether effects of decisions on citizens’ lives are produced by punitive or assistive actions. This distinction has been recently identified as a crucial factor in the selection of fairness notions for ML applications^14^^,^^15^ and because individuals may differ in their perception of the severity of these types of decisions (see background and related work). This distinction allows us to investigate different kinds of decisions within identical contexts. The kinds of *data* used for decision-making have been a key concern of previous empirical research on fairness perceptions. Although previous studies usually focus on specific kinds of information to be used, we follow the notion of contextual integrity,^27^ which suggests that the crucial question is whether the use of the data is contextually appropriate (see background and related work). We distinguish between contextually close and contextually remote kinds of data for each context. For instance, contextually close data in the hiring context may be data on performance in previous jobs. Across all investigated contexts, contextually remote data may be data from Internet searches about a person who, e.g., applies for credit. The latter data might improve the accuracy of decisions, but privacy concerns about the appropriateness of their use may arise, particularly if the data in question are not necessarily related to the decision problem at hand. For our purposes, it does not matter which exact kind of additional (Internet) data is considered, what is important is that these data are potentially considered as out of context by respondents but may still improve the accuracy of predictions. Finally, we vary the degree of human involvement in the decision-making process (*decision-maker*) to learn about its optimal levels across different contexts, which represents one of the most crucial design decisions for computationally supported decision-making systems. The concrete levels for each of the dimensions are as follows:

1. Type of action the decision affects (dimension: *action*)
  • Assistive action
    – Bank: provision of exclusive financial products
    – Job: hiring of employees
    – Prison: early release from prison
    – Unemployment: offering support services to unemployed individuals
  • Punitive action
    – Bank: regulating access to credits
    – Job: termination of work in probation period
    – Unemployment: shortening financial assistance for unemployed individuals
    – No punitive action was defined for the justice context because we deemed this case too problematic to confront respondents
2. Type of data used to inform decision (dimension: *data*)
  • Only data that have been produced in the social context of the decision task or closely related contexts (“no Internet data”)
  • Additionally using data found on the Internet that may stem from various contexts (“Internet data”)
3. Who makes the decision (dimension: *decision-maker*)
  • Solely ADM (fully automated: “Algorithm”)
  • Human decision-making based on an automated recommendation (automated recommendation: “Both”)
  • Solely human decision-making, assisted by information from computer programs (mainly human: “Human”)

For instance, the vignette with the levels employment agency, assistive action, additional Internet data, and mainly human decision-making reads: “A local employment agency has developed a computer program for assigning support measures to job seekers. This program uses data about the person’s past periods of employment and unemployment, as well as information about the person available on the Internet. A staff member at the employment agency compares this information with that of other job-seeking individuals who have successfully participated in a measure. The employee decides whether the person is to receive a support measure” (translated from German).

In the vignette with the levels employment agency, assistive action, and additional Internet data, but automated recommendation, the last two sentences above are changed as follows: “The program compares this information with that of other job-seeking individuals who have successfully participated in a measure. The program gives an employee a recommendation whether the person is to receive a support measure. The final decision is made by the employee.”

In the corresponding vignette with fully ADM, the last two sentences read: “The program compares this information with that of other job-seeking individuals who have successfully participated in a measure. The program determines automatically whether the person is to receive a support measure.”

All vignettes are presented in the data documentation of Wave 54 of the GIP^43^ and in the supplemental experimental procedures.

After each vignette, we asked respondents in two separate questions how fair and how acceptable they perceive this way of decision-making (“How fair do you find it is to make a decision in this way?” “How acceptable do you find it is to make a decision in this way?”) using a fully labeled four-point rating scale (“Not at all fair/acceptable,” “A little fair/acceptable,” “Somewhat fair/acceptable,” or “Very fair/acceptable”). We ask about both fairness perceptions and acceptability because the former may be only one among various factors that affect acceptance. In addition to fairness, individuals may consider accountability, transparency, and explainability in their overall assessment of algorithmic decision-making, next to their evaluation of the systems utility.^44^ Thus, individuals may think that a system is prone to producing unfair results but still be convinced that the system is transparent or more efficient and therefore acceptable. Note that we do not force individuals into a specific role in the ADM process (such as a decider or an affected individual) to learn about citizens’ evaluations of the systems as such.

Note that we refrained from pre-defining fairness (or acceptability) for the respondents in our survey instrument. Our aim was to measure respondents’ personal perception of the general appropriateness of the presented way of decision-making, without priming and limiting them toward a specific (technical) fairness notion that they might not even consider in real-world evaluations of ADM.

### Respondent characteristics

In addition to fairness and acceptability evaluations, we collected information on respondents’ socio-demographic characteristics and further background information. We are therefore able to study how fairness perceptions depend on respondents’ gender (male and female) and age (older than 60 years versus 60 years or younger). Similar to other countries, these two individual attributes are oftentimes connected to discrimination in Germany.^45^ In line with the treatment of these characteristics as protected attributes in the fairness literature, this allows us to investigate whether historical disadvantages may be associated with differential fairness evaluations of ADM systems across social groups. We further constructed a “privacy” index that summarizes respondents’ concerns toward sharing personal data on a five-point scale (labeled from “not at all concerned” to “very concerned”), one measure that aims at capturing general affinity toward technology (via the total number of digital devices owned) and one measure to assess respondents’ knowledge of algorithmic decision-making (via the total number of specific technical and statistical terms known; see Table S2 for details). These variables allow us to investigate whether ADM design features are evaluated differently given individuals’ privacy attitudes and technical experience.

### Analysis

We conduct our analysis in three steps. First, we present descriptive findings of the fairness evaluations by vignette dimensions. Second, we show results of mixed-effects ordinal probit regressions that model the effects of the ADM’s application context and design dimensions on fairness and acceptability assessments. Third, we present context-specific regression models that investigate the effects of respondent characteristics. We use mixed-effects models to account for the hierarchical structure of our data, because multiple (four) vignettes are nested within respondents.^46^ For our fairness measure, e.g., this gives us 15,525 observations based on 3,930 respondents. Given the four ordered response categories of the outcome variables, we follow an ordinal probit approach by linking the observed outcome to an unobserved, continuous response variable via a set of threshold functions.^47^ In our mixed-effects models, we include random intercepts on the respondent level and specify different model variations, including random slopes, to test our assumptions about the mechanisms of fairness perceptions. All regression models control for the order of vignettes shown to respondents to eliminate ordering effects.

## Results

### Distribution of fairness evaluations

**Figure 1:**
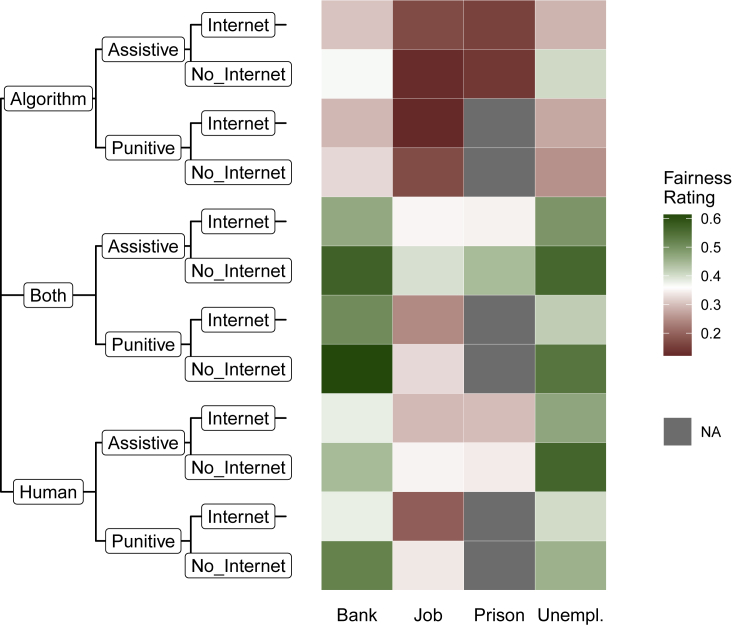
Average fairness rating by vignette levels The heatmap shows relative frequencies of respondents that rated a scenario as “Fair” (i.e., either “Somewhat fair” or “Very fair”). The color scale is centered at the average fairness rating over all vignettes.

We first present average fairness ratings depending on vignette characteristics to provide a straightforward overview of the main results. For interpretation purposes, we collapse the four-point response scale into two categories: “Fair” (“Somewhat fair” and “Very fair”) and “Not fair” (“A little fair” and “Not at all fair”) and show the relative frequencies of respondents that rated a scenario as “Fair” in Figure 1. A tabular presentation of relative frequencies for both fairness and acceptance ratings by vignette levels is provided in Table S1. Overall summary statistics for fairness and acceptance evaluations, as well as for respondent characteristics, are provided in Table S2. A comparison of fairness ratings across vignettes allows the following four conclusions.

First, the highest response categories (“Somewhat fair” and “Very fair”) were less frequently chosen than “A little fair” and “Not at all fair,” indicating some, although not strong, levels of skepticism against computationally supported decision-making on average. Nonetheless, the level of perceived unfairness strongly depends on the specific vignette characteristics.

Second, fairness evaluations vary by application *context*. In particular, the use of ADM in HR contexts (vignette level “Job”) and criminal justice settings (“Prison”) is often evaluated as “Not at all fair” or “A little fair,” whereas ADM applications in the banking sector (“Bank”) or by employment agencies (“Unemployment”) are perceived as less troubling.

Third, decisions performed without any kind of human intervention (“Algorithm”) are perceived as less fair than decisions that include human supervision (“Both” and “Human”). These differences along the dimension *decision-maker* are strongly pronounced for the HR and judicial context, considering their low baseline levels.

Fourth, within contexts, respondents do not appear to strongly distinguish between punitive and assistive *actions*. However, a slight shift toward higher perceived fairness is observable for ADM scenarios that do not use Internet *data*.

We present descriptive results of both the (complete) fairness and acceptance evaluations, including all response categories in Figures S1 and S2. Overall, the acceptance evaluations show very similar patterns as the fairness ratings, indicating that respondents evaluated fairness primarily with respect to whether they find the presented way of decision-making appropriate (in a given context). This result may also mean that a common latent construct underlies these two measures. We can, however, notice that respondents are somewhat more restrictive in their acceptability ratings, because the highest response category (“Very acceptable”) was rarely chosen across vignettes.

### Mixed-effects regression models

**Figure 2:**
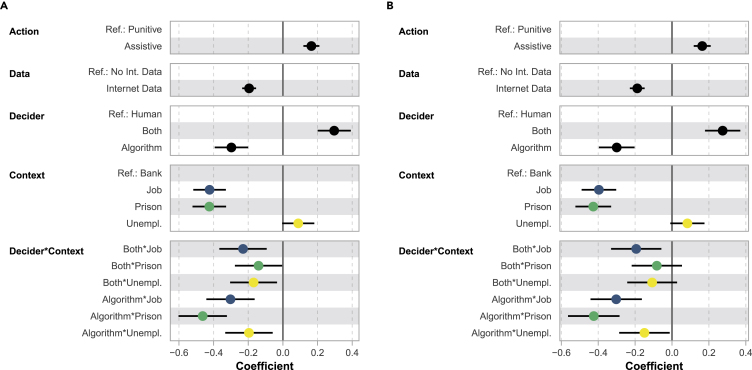
Coefficients (with 95% confidence intervals) of mixed-effects ordinal probit regression models predicting fairness evaluations and acceptance ratings with interactions between vignette dimensions *decision-maker* and *context* (R-I Interaction) (A) Outcome: fairness (nObs = 15,525). (B) Outcome: acceptance (nObs = 15,566).

We fitted three mixed-effects regression models for each outcome variable, i.e., respondents’ fairness evaluations and acceptance ratings: a random-intercept model with main effects of all vignette dimensions (R-I Main), a random-intercept model with additional interactions between the dimensions *decision-maker* and *context* (R-I Interaction), and a random-intercept-random-slope model that allows the effects of *decision-maker* to vary between respondents (R-I-R-S). Focusing on the interactions between *decision-maker* and *context* allows us to shed light on how crucial ADM design decisions drive contextual fairness evaluations and add to the (in part inconclusive) research on publicly accepted degrees of automation in different application settings. Because the interactions are of most substantive interest, we present the R-I Interaction model for both outcome variables in Figure 2. Model fit statistics and tests for all models are summarized in Table S3.

The results of the R-I Interaction model predicting fairness evaluations (Figure 2A) point to the following conclusions: computationally supported decision-making systems that inform assistive *actions* are perceived as fairer than their punitive counterparts. Applications that make additional use of Internet *data* are perceived as less fair, compared with systems that only draw on contextually related data. The conditional main effects of *decision-maker* show that automated recommendation (“Both”) is perceived as fairer and fully ADM (“Algorithm”) as less fair compared with mainly human decision-making (in the “Bank” context). We further see that respondents valued a stronger human component in the “Job,” “Prison,” and “Unemployment” context as indicated by the negative interaction effects of *decision-maker* with *context*. Strong negative interactions for fully ADM with the “Job” and “Prison” *context* can be observed (“Algorithm∗Job”, “Algorithm∗Prison”). Starting from already negative conditional main effects, the results for “Job” and “Prison” show that ADM is perceived as particularly problematic in these settings.

To ease interpretation, we present average predicted probabilities for all outcome categories based on the R-I Interaction model across vignette dimensions in Table S4. We see that differences in the predicted probabilities of a positive fairness assessment (“Somewhat fair” and “Very fair”) are driven by the vignette dimensions *context* and *decision-maker*, with considerably higher average predicted probabilities of both (highest) outcome categories for automated recommendation and the “Bank” and “Unemployment” settings. Focusing on the interaction effects, Table S5 shows how differences in the predicted probabilities across levels of *decision-maker* vary by *context*, highlighting that the distance between “Algorithm” versus “Human” is particularly strong in the “Job,” “Prison,” and “Unemployment” context (for the response categories “Somewhat fair” and “Not at all fair”).

Comparing the outlined model with interactions against a model that includes only main effects underlines the context dependency of fairness perceptions, because the former model results in a considerably better model fit (likelihood ratio test of R-I Interaction versus R-I Main; see second column in Table S3). An increase in model fit can also be observed when specifying random slopes for *decision-maker*, indicating that the effects of this vignette dimension vary between respondents (likelihood ratio test of R-I-R-S versus R-I Main; see last column in Table S3). These findings motivate the specification of context-specific regression models that include interactions between the dimension decision-maker and respondent characteristics.

The results of the mixed-effects models predicting acceptance ratings mirror the above findings. The corresponding R-I Interaction model (Figure 2B) shows almost identical effect patterns: computationally supported decision-making is deemed less acceptable in the “Job” and “Prison” context (compared with “Bank”) and respondents particularly object to fully ADM in these settings. We also note that for both outcomes we observe intra-class correlations (ICCs) between 0.45 and 0.51, highlighting that there is considerable clustering of vignette ratings within respondents (Table S3 again).

### Context-specific regressions

**Figure 3:**
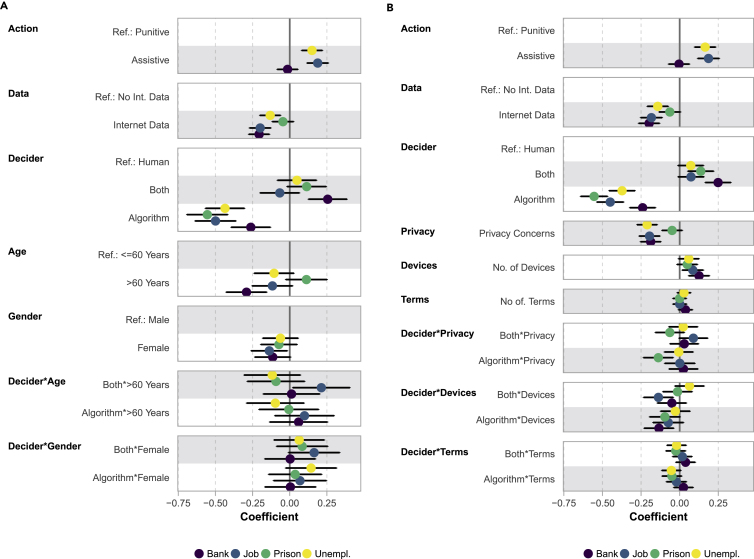
Coefficients (with 95% confidence intervals) of ordinal probit regression models predicting fairness evaluations of each *context* with interactions between the vignette dimension *decision-maker* and respondent characteristics (A) Context-specific Interactions 1 (nBank = 3,653, nJob = 3,660, nPrison = 3,652, nUnempl = 3,654). (B) Context-specific Interactions 2 (nBank = 3,854, nJob = 3,858, nPrison = 3,855, nUnempl = 3,851).

We present two sets of context-specific regression models that include both vignette and respondent characteristics in Figure 3. The first set includes respondents’ age and gender, in interaction with the vignette dimension *decision-maker*. The second set of models includes measures of respondents’ privacy concerns, the number of digital devices owned, and the number of technical terms known (reflecting familiarity with AI and ML), all in interaction with *decision-maker*. Each set consists of four regression models that were fitted separately to fairness evaluations of each *context*. Corresponding models for the outcome acceptance are shown in Figure S3.

The results of the first model set (Figure 3A) show a negative conditional main effect of age in the “Bank” context, indicating that, in this case, older respondents perceive computationally supported decision-making as less fair than younger respondents. We generally observe little effect differences regarding the vignette dimension decision-maker between older and younger respondents. A notable exception is the more positive evaluation of automated recommendation of older respondents (“Both∗ >. 60 Years”) in the “Job” context. We do not observe strong differences in the evaluation of either type of decision-making based on gender. At most, a modestly lower fairness evaluation of computationally supported decision-making of female respondents can be observed in the “Job” context (conditional main effect of gender).

Model set two (Figure 3B) shows negative conditional main effects of respondents’ privacy concerns in the “Bank”, “Job,” and “Unemployment” contexts. Computationally supported decision-making is particularity viewed as problematic by people with higher privacy concerns. For the “Prison” context, stronger worries about privacy coincide with a more negative evaluation of fully ADM (“Algorithm∗Privacy”). Respondents’ affinity toward technology seems to play a minor role in shaping fairness evaluations of ADM systems. Nonetheless, we can observe positive conditional main effects of the number of digital devices owned by respondents on fairness evaluations in the “Bank” and “Job” contexts and negative interactions between devices and fully ADM (“Algorithm∗Devices”) and automated recommendation (“Both∗Devices”) in selected settings.

The results of the context-specific regression models predicting acceptance ratings show similar results, although with some exceptions, particularly in the first model set (Figure S3A). This includes an additional negative conditional main effect of age in the “Job” context and higher acceptance ratings of fully ADM of female compared with male respondents in the “Unemployment” context.

## Discussion

In this research, we set out to advance our understanding of perceptions of fairness of ADM systems. Specifically, we sought to measure how design decisions, such as the level of human involvement in making the final decision and characteristics of the decision itself (assistive versus punitive), as well as the type of scenario, impact acceptance of various ADM systems and their perceived fairness. Our results provide implications for how to design ADM applications depending on context. Furthermore, they offer insights into acceptance of assistive and punitive types of decisions across contexts, showing in which cases human involvement should be particularly considered in ADM design. A variation in the scenarios considered, in combination with a nationally representative probability-based sample of the German population, allows us to draw conclusions that future research may use as a starting point to understand the mechanisms causing variation in fairness evaluations across contexts.

### Context dependency

Overall, the perceived fairness of computationally supported decision-making varies across contexts of application. Fairness ratings are lower in the “Job” and “Prison” contexts than in the “Bank” and “Unemployment” contexts. We believe that individuals may be particularly sceptical about automation in high-stake contexts (such as the “Prison” scenario) and in settings that may both eventually affect themselves and can have considerable impact (as in the “Job” context) as theories of subjective expected utility^48^ suggest. However, we note that we did not measure subjective evaluations of impact; thus, we can only speculate that the perceived impact of a decision (e.g., high stakes versus low stakes) may cause the differences between these contexts.

Furthermore, we find that *assistive* decisions are deemed fairer than punitive decisions in the “Job” and “Unemployment” context, while no such difference is found in the “Bank” context. Following prospect theory,^16^ individuals may weigh potential losses higher than potential gains and therefore be more open to assistive decisions. In our vignettes, the change in stakes from assistive to punitive decision-making in contexts that are related to hiring and the labor market are potentially perceived higher than in the “Bank” context. Regarding the implications of this finding for the design of ADM systems, we believe that fairness should be a major concern when the impact of the decision is high and the decision is rather punitive than assistive. However, future research will have to dig deeper into the underlying dimensions of contexts that affect human perceptions of ADM systems.

### Human involvement

A second central finding concerns the comparison of fairness ratings for different degrees of human involvement in decision-making: respondents on average deemed automated recommendations as fairer than fully ADM and as similarly fair as mainly human decision-making. This finding suggests that individuals do not consider the use of algorithms to inform decision-making as necessarily problematic per se. However, at the same time, respondents value the involvement of humans in the decision-making process. Therefore, human oversight appears to be an important element to ameliorate fairness perceptions of the population. While previous literature has shown such tendencies in specific contexts,^21^ we show how this effect varies across contexts. In our data, this is particularly true for the “Job” and “Prison” contexts, which are the two contexts in which computationally supported decision-making is generally perceived to be less fair than in the other contexts (see above). That is, ADM applications that may already be perceived as requiring special attention may deserve more human involvement in the decision-making process in order to be perceived as fair. Challenges with trust in novel technologies and misperceptions of the technological risk (e.g., to be treated unfair) may be important drivers for a desire of human oversight. Therefore, designing ADM systems that are perceived as fair may require effective communication of a basic understanding of the underlying technology. Moreover, individuals may feel more comfortable if high-stake decisions, especially in punitive contexts, involve a certain degree of human involvement or oversight in the decision-making process. Finally, if the automated element in decision-making itself is given a human appearance, it may enjoy increased acceptance, as previous research on chatbots suggests.^49^

Previous research suggests that higher complexity of the decision task is connected to higher fairness ratings for human versus algorithmic decision-making.^17^ Our finding that human involvement is particularly desired in the hiring context aligns with a previous study in which respondents on average deemed human managers as fairer decision-makers for hiring decisions than algorithms.^18^ Lee^18^ also draws on open-ended responses, showing that this result may be based on expectations of human managers’ skills and the concern that algorithms took a too standardized approach to evaluate candidates. It is possible that decisions relating to banking and unemployment are considered to be more amenable to standardization than decisions relating to hiring and prisons.

### Data used in ADM

In our study, respondents perceived systems that draw on additional Internet data for decision-making less fair than systems that relied only on data that are close to the respective context. This finding is in line with previous research on feature use in ADM systems (see background and related work). It confirms the importance of appropriate information flow, central to the privacy theory of contextual integrity.^27^ Contextual integrity emphasizes that social contexts shape privacy norms, i.e., whose and which data are appropriate to be transmitted under which conditions.

### Individual characteristics

As for the impact of individual socio-demographic characteristics, general fairness ratings of the “Bank” context decrease with higher age, and ratings are lower for women than for men in the “Job” context. Although the uncertainty in the estimated coefficients should make us cautious in over-interpreting these findings, they may hint to the presence of self-interest and/or social identity effects in fairness perceptions and could be worth exploring further. Previous research suggests that there appears to be self-interest involved in the individual evaluation of ADM processes and feature use.^28^^,^^50^ Another potential theoretical explanation follows the idea of social identity theory.^51^ That is, individuals may not accept those decisions that may harm their in-group.^52^ Applied to the present study, these perspectives would imply that older people and women may consider that they or their in-group may be particularly disadvantaged in bank- or job-related contexts, respectively. This finding appears to be unrelated to the degree of human involvement. Furthermore, as previous research suggests, placing respondents into a specific position in the described decision-making process (such as decider or being affected by the decision oneself) may lead to different responses.^53^

### Fairness versus acceptance

The regression results for the second investigated outcome variable “acceptance” mostly mirror the findings on fairness perceptions, although with some exceptions in the context-specific regressions. Indeed, the Spearman rank correlation coefficient for these two variables is 0.907. Although we cannot rule out that these similarities are a result of problematic respondent behavior (i.e., it could be possible that some respondents use satisficing strategies^54^ when responding to the survey questions), it is conceivable that fairness and acceptance presuppose each other in evaluations of ADM systems, or that they measure a common latent construct. This latent construct may reflect an overall notion that using the respective ADM system is “okay” or desirable.

### Limitations of the study

The study presented here draws on a very carefully selected sample of the German population. However, the vignette task used here for measurement is complex, and it is possible that not all respondents fully understood all questions and settings. Ideally, we would have been able to add on qualitative interviews to capture why people responded the way they did and what exactly they thought about when reading about algorithms. Such probing questions are uncommon in fully standardized interviews and would have not been possible in this data collection instrument.

We also note that in measuring respondents’ fairness perceptions, we cannot infer which notion(s) of fairness they operationalize in their evaluations. Respondents may consider notions of disparate treatment or impact with respect to attributes that they may perceive as sensitive or protected, or they may envision differential prediction (and thus decision) errors^55^ as a result of a specific ADM design. Most likely, fairness assessments are the result of a (weighted) combination of multiple dimensions, which also are dependent on the presented ADM application context. Additional research is needed to probe which fairness concepts respondents may consider as most relevant in a given context.

Although we tried to capture a set of relevant contexts and settings, the study does not cover all possibly varying design characteristics of ADM systems. Previous studies have drawn on a plethora of potentially relevant characteristics, and these should also be considered when designing concrete ADM systems. Our intention was not to evaluate concrete ADM systems in detail but to compare crucial design elements within and between contexts of application, with an emphasis on the particularly important element of who makes the final decision and which kind of decision (assistive or punitive) is to be taken. Although we believe that the potential impact of a decision plays an important role in fairness evaluations, we did not directly manipulate whether a decision is high or low stakes. Therefore, we can only speculate that the potential impact of a decision will be a decisive element in individuals’ fairness evaluations of ADM systems.

### Future work

To expand the generalizability of our findings, future research may consider additional contexts and more nuances of the decision-making process. This may include a systematic variation of the complexity and the potential impact (high versus low stakes) of a decision, as well as the degree to which a decision is perceived to require human skills, such as subjective and intuitive judgment (see also background and related work). Furthermore, previous research has shown that the exact wording with which the computerized components of ADM systems are described affect perceptions,^56^ which may be particularly interesting to compare across further contexts. This may also include surveying populations in other countries than Germany and a focus on specific, potentially disadvantaged populations. This would allow researchers to investigate the impact of further protected attributes, such as ethnicity, on fairness evaluations. Such research could be conducted in real-life settings or with more immediate, real scenarios to verify the external validity of our findings.

More importantly, however, future work may put special emphasis on cleanly identifying the underlying dimensions that affect human perceptions of ADM systems. For example, a generalizable model of the influence of dimensions on fairness evaluations would allow policy-makers to estimate the degree to which a planned ADM system will meet society’s normative expectations. Such a model should include understanding the mechanisms that cause variation in fairness perceptions, and integrate them in a theoretical model, a point also raised by Langer and Landers.^21^ Right now, we can only speak to the dimensions that we experimentally varied in our study. In summary, we recommend that applications used to inform punitive decisions, applications with no human involvement, and applications that are not fully transparent regarding the data used should be carefully designed because fairness concerns among individuals seem to be highest in these scenarios.

### Conclusion

In conclusion, our study showed that respondents perceive a combination of human and algorithmic decision-making as acceptable as decisions made by a human decider only. Solely algorithmic decisions are less accepted in the instances examined here. Human oversight is therefore deemed a desirable element of ADM systems. Overall, we found fairness perceptions not to be very high but to vary notably across context and design features.

There is a variety of decision tasks we did not touch on. Neither did we investigate perceptions of biometric mass surveillance, drones, and related situations with even higher stakes, nor did we investigate very low-stakes decisions such as algorithm-based navigation suggestions. Even within this narrower scope we see variation in perceptions, driven by context and type of decision, the used data, and individual characteristics. These attitudes are likely to shift with societies becoming more exposed to a variety of ADM systems. For now we want to re-emphasize that context matters, and individual preferences should be taken into consideration when designing these systems. Mapping novel ADM systems along the dimensions that we tested in this study may inform ADM designers beforehand when and where fairness concerns may arise among those impacted by the decisions.

## Experimental procedures

### Resource availability

#### Lead contact

For any questions regarding the paper and resources, please contact Dr. Christoph Kern (c.kern@uni-mannheim.de).

#### Materials availability

This study did not generate new unique reagents.

## Data Availability

The questionnaire and the data have been deposited at data archive GESIS: https://doi.org/10.4232/1.13835 and are publicly available as of the date of publication. Application and written permission are needed prior to data access through the archive. All original code has been deposited at OSF: https://doi.org/10.17605/OSF.IO/W645F and is publicly available as of the date of publication. Any additional information required to reanalyze the data reported in this paper is available from the lead contact on request.

## Supplemental information

Document S1. Figures S1–S3, Tables S1–S5, and supplemental experimental procedures

Document S2. Article plus supplemental information
