# Supplementary material for: Humans versus machines: Who is perceived to decide fairer? Experimental evidence on attitudes toward automated decision-making
Source: Patterns (N Y). 2022 Sep 29;3(10):100591. doi: 10.1016/j.patter.2022.100591 (PMC9583126; doi:10.1016/j.patter.2022.100591)
Supplement: Document S1. Figures S1–S3, Tables S1–S5, and supplemental experimental procedures [file mmc1.pdf]

Patterns, Volume *3*

Supplemental information

Humans versus machines: Who is perceived to decide fairer? Experimental evidence on attitudes toward automated decision-making

Christoph Kern, Frederic Gerdon, Ruben L. Bach, Florian Keusch, and Frauke Kreuter

A. Supplemental Results

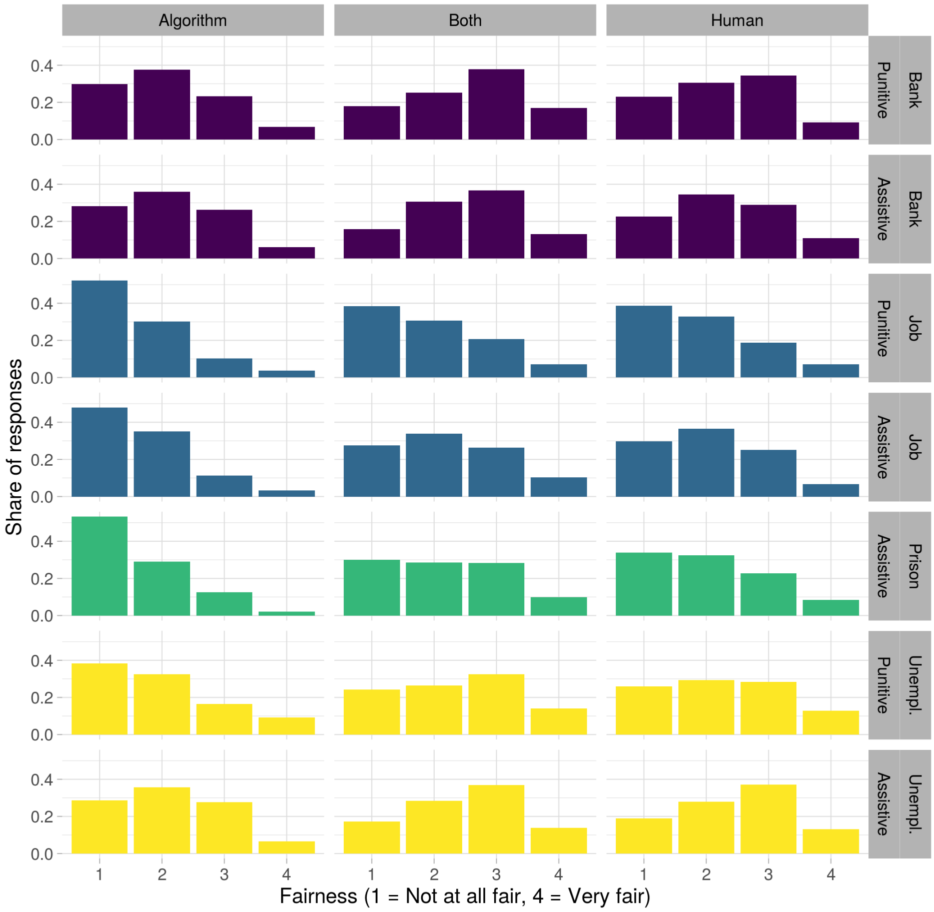

Figure S1: Distribution of fairness evaluations by vignette levels.

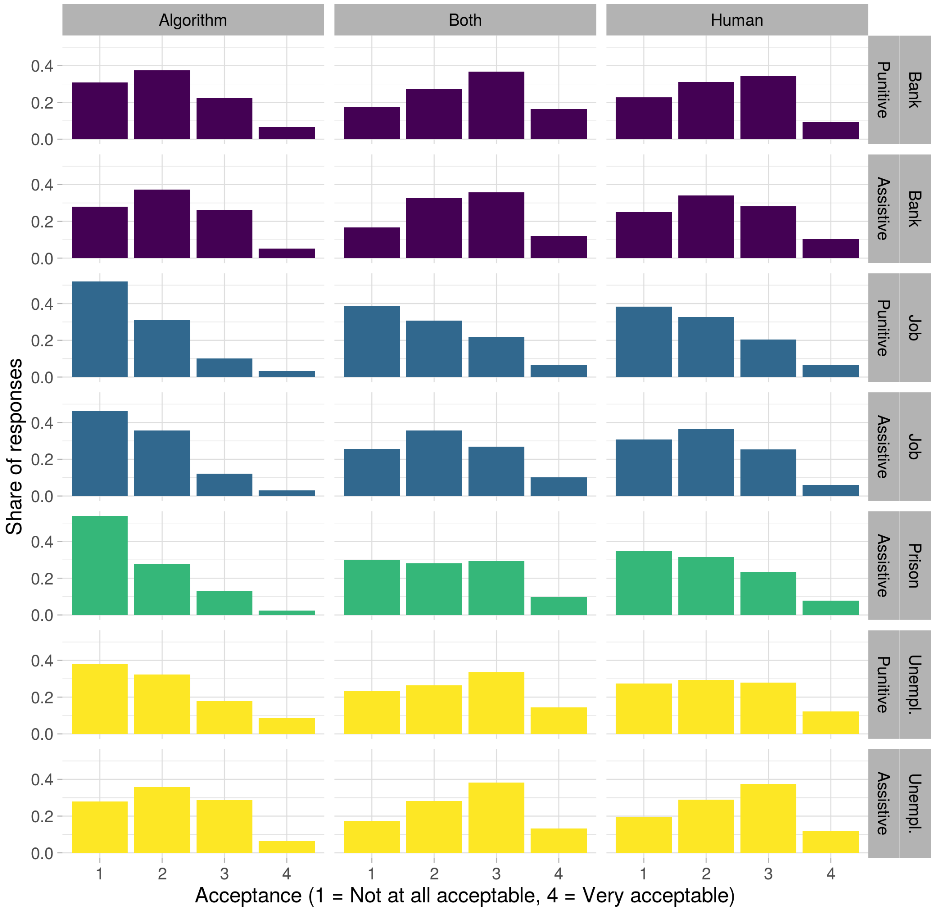

Figure S2: Distribution of acceptance ratings by vignette levels.

(a) Context-specific Interactions 1 ($n_{Bank} = 3662$, $n_{Job} = 3671$, $n_{Prison} = 3660$, $n_{Unempl.} = 3666$)

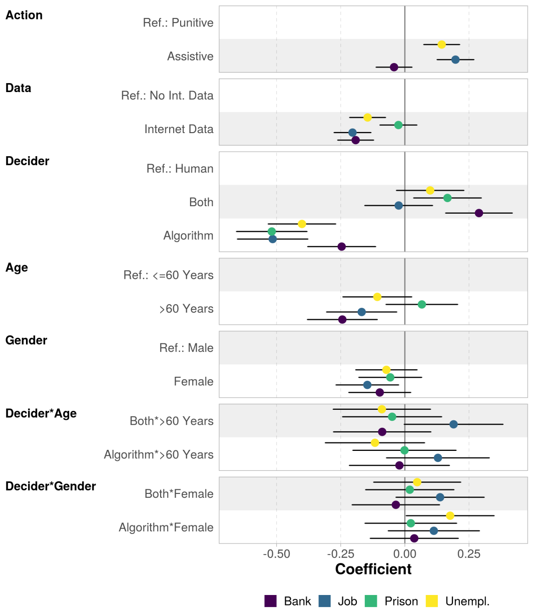

(b) Context-specific Interactions 2 ($n_{Bank} = 3852$, $n_{Job} = 3855$, $n_{Prison} = 3852$, $n_{Unempl.} = 3854$)

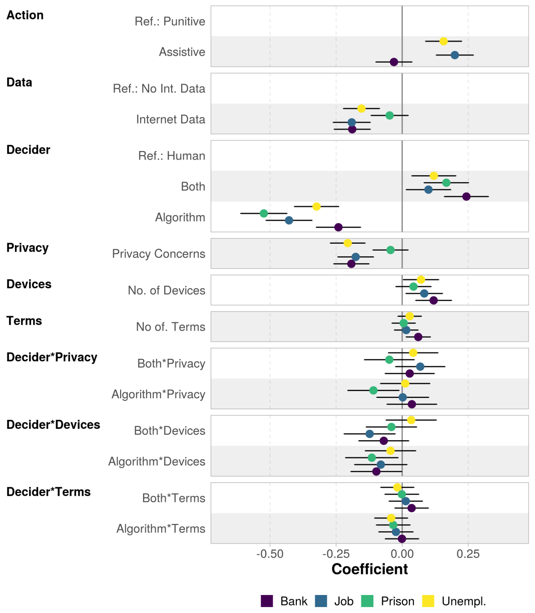

Figure S3: Coefficients (with 95% confidence intervals) of ordinal probit regression models predicting acceptance ratings of each *context* with interactions between the vignette dimension *decision-maker* and respondent characteristics.

Table S1: Average fairness and acceptance ratings by vignette levels.

(a) Relative frequencies of respondents that rated a scenario as "Fair" ("Somewhat fair" or "Very fair").

			Context			
Decision-maker	Action	Data	Bank	Job	Prison	Unempl.
Algorithm	Assistive	Internet	0.31	0.17	0.16	0.29
Algorithm	Assistive	No Internet	0.37	0.13	0.14	0.40
Algorithm	Punitive	Internet	0.29	0.12		0.28
Algorithm	Punitive	No Internet	0.32	0.17		0.25
Both	Assistive	Internet	0.47	0.35	0.35	0.49
Both	Assistive	No Internet	0.57	0.40	0.44	0.56
Both	Punitive	Internet	0.50	0.25		0.42
Both	Punitive	No Internet	0.61	0.33		0.54
Human	Assistive	Internet	0.38	0.30	0.30	0.47
Human	Assistive	No Internet	0.44	0.35	0.34	0.56
Human	Punitive	Internet	0.38	0.20		0.40
Human	Punitive	No Internet	0.52	0.34		0.45

(b) Relative frequencies of respondents that rated a scenario as "Acceptable" ("Somewhat acceptable" or "Very acceptable").

			Context			
Decision-maker	Action	Data	Bank	Job	Prison	Unempl.
Algorithm	Assistive	Internet	0.29	0.15	0.17	0.30
Algorithm	Assistive	No Internet	0.36	0.16	0.15	0.41
Algorithm	Punitive	Internet	0.29	0.10		0.29
Algorithm	Punitive	No Internet	0.31	0.17		0.26
Both	Assistive	Internet	0.44	0.34	0.37	0.49
Both	Assistive	No Internet	0.54	0.41	0.44	0.57
Both	Punitive	Internet	0.50	0.26		0.42
Both	Punitive	No Internet	0.58	0.32		0.56
Human	Assistive	Internet	0.35	0.30	0.30	0.46
Human	Assistive	No Internet	0.43	0.34	0.34	0.55
Human	Punitive	Internet	0.39	0.20		0.39
Human	Punitive	No Internet	0.50	0.34		0.44

Table S2: Summary statistics.

(a) Vignettes

Variable	Values	Freqs (% of Valid)	Valid
Fairness	Not at all fair	5058 (32.6%)	15525 (97.2%)
	A little fair	5016 (32.3%)	
	Somewhat fair	4036 (26.0%)	
	Very fair	1415 (9.1%)	
Acceptance	Not at all acceptable	5078 (32.6%)	15566 (97.5%)
	A little acceptable	5044 (32.4%)	
	Somewhat acceptable	4101 (26.3%)	
	Very acceptable	1343 (8.6%)	

(b) Respondents

Variable	Stats/ Values	Freqs (% of Valid)	Valid
Age	≤60 Years	2729 (72.6%)	3760 (94.2%)
	>60 Years	1031 (27.4%)	
Gender	Male	2072 (51.9%)	3991 (99.9%)
	Female	1919 (48.1%)	
Number of Devices†	Mean (sd) : 2.6 (0.9)	0 : 25 (0.6%)	3876 (97.1%)
	min < med < max:	1 : 335 (8.6%)	
	0 < 3 < 5	2 : 1379 (35.6%)	
	IQR (CV) : 1 (0.3)	3 : 1452 (37.5%)	
		4 : 636 (16.4%)	
		5 : 49 (1.3%)	
Number of Terms††	Mean (sd) : 3.3 (1.4)	0 : 184 (4.7%)	3877 (97.1%)
	min < med < max:	1 : 266 (6.9%)	
	0 < 3 < 5	2 : 491 (12.7%)	
	IQR (CV) : 2 (0.4)	3 : 1046 (27.0%)	
		4 : 900 (23.2%)	
		5 : 990 (25.5%)	
Privacy Index†††	Mean (sd) : 3.5 (0.9)		3873 (97.0%)
	min < med < max:		
	1 < 3.5 < 5		
	IQR (CV) : 1 (0.3)		

†Answer categories: Smartphone, Cell Phone, Desktop Computer, Tablet, eBook Reader.

††Answer categories: Artificial Intelligence, Computer Algorithms, Machine Learning, Recommender Systems, Targeted/personalized Ads.

†††Item 1: "I do not mind sharing personal information as nowadays everyone is doing this anyway."

Item 2: "You cannot live in the modern world without sharing personal information."

Item 3: "When you provide personal information you never know who else is going to see it."

Item 4: "I do not mind sharing personal information in return for a product or service that I want."

Table S3: Random effects estimates and model fit indices of mixed-effects ordinal probit regression models predicting fairness evaluations and acceptance ratings.

(a) Outcome: Fairness

	R-I Main	R-I Interaction	R-I-R-S
LL	−18 234.01	−18 206.79	−18 196.84
BIC	36 583.83	36 587.28	36 557.73
ICC	0.46	0.46	0.51
Variance: Intercept	0.84	0.84	1.04
Variance: Decider Both			0.09
Variance: Decider Algorithm			0.38
L-R Test		†54.45	†74.35
Num. Observations	15 525	15 525	15 525
Num. Respondents	3930	3930	3930

†: $p \leq 0.001$

(b) Outcome: Acceptance

	R-I Main	R-I Interaction	R-I-R-S
LL	−18 227.38	−18 202.31	−18 188.81
BIC	36 570.59	36 578.36	36 541.71
ICC	0.45	0.45	0.50
Variance: Intercept	0.83	0.83	1.02
Variance: Decider Both			0.07
Variance: Decider Algorithm			0.43
L-R Test		†50.14	†77.14
Num. Observations	15 566	15 566	15 566
Num. Respondents	3972	3972	3972

†: $p \leq 0.001$

Table S4: Average predicted probabilities based on the R-I Interaction model. Predictions for a given predictor level are computed while setting the remaining vignette dimensions to their reference level.

(a) Outcome category "Very fair"

Predictor	$\hat{P}(y=4)$	95% CI	
Bank	0.06	0.06	0.07
Hire	0.02	0.02	0.02
Prison	0.02	0.02	0.02
Unempl	0.06	0.05	0.07
Human	0.04	0.04	0.05
Both	0.06	0.06	0.07
Alg.	0.02	0.01	0.02
Punitive	0.03	0.03	0.04
Assistive	0.05	0.04	0.05
No Internet	0.05	0.04	0.05
Internet	0.03	0.03	0.04

(b) Outcome category "Somewhat fair"

Predictor	$\hat{P}(y=3)$	95% CI	
Bank	0.34	0.32	0.35
Hire	0.19	0.18	0.20
Prison	0.19	0.17	0.20
Unempl	0.33	0.31	0.34
Human	0.29	0.27	0.30
Both	0.33	0.31	0.34
Alg.	0.16	0.15	0.17
Punitive	0.24	0.23	0.25
Assistive	0.28	0.27	0.29
No Internet	0.28	0.27	0.29
Internet	0.24	0.23	0.25

(c) Outcome category "A little fair"

Predictor	$\hat{P}(y=2)$	95% CI	
Bank	0.41	0.40	0.42
Hire	0.41	0.40	0.43
Prison	0.40	0.39	0.41
Unempl	0.41	0.40	0.43
Human	0.43	0.42	0.44
Both	0.41	0.40	0.42
Alg.	0.39	0.38	0.40
Punitive	0.41	0.40	0.42
Assistive	0.41	0.40	0.42
No Internet	0.41	0.40	0.42
Internet	0.41	0.40	0.42

(d) Outcome category "Not at all fair"

Predictor	$\hat{P}(y=1)$	95% CI	
Bank	0.19	0.17	0.20
Hire	0.38	0.36	0.40
Prison	0.39	0.37	0.41
Unempl	0.20	0.18	0.21
Human	0.24	0.23	0.25
Both	0.20	0.18	0.21
Alg.	0.43	0.41	0.45
Punitive	0.31	0.30	0.33
Assistive	0.26	0.25	0.28
No Internet	0.26	0.25	0.27
Internet	0.32	0.31	0.33

Table S5: Average conditional predicted probabilities based on the R-I Interaction model. Predictions for a given level of *decision-maker* are computed conditional on different levels of *context*, while setting the remaining vignette dimensions to their reference level.

(a) Outcome category "Very fair"

Context	Decider	$\hat{P}(y=4)$	95% CI	
	Human	0.07	0.06	0.08
Bank	Both	0.12	0.10	0.13
	Alg.	0.04	0.03	0.04
	Human	0.03	0.02	0.03
Job	Both	0.03	0.03	0.04
	Alg.	0.01	0.00	0.01
	Human	0.03	0.02	0.03
Prison	Both	0.04	0.03	0.05
	Alg.	0.00	0.00	0.00
	Human	0.08	0.07	0.09
Unempl.	Both	0.10	0.09	0.11
	Alg.	0.03	0.02	0.03

(b) Outcome category "Somewhat fair"

Context	Decider	$\hat{P}(y=3)$	95% CI	
	Human	0.36	0.34	0.38
Bank	Both	0.43	0.41	0.45
	Alg.	0.28	0.26	0.30
	Human	0.25	0.22	0.27
Job	Both	0.26	0.24	0.28
	Alg.	0.11	0.09	0.12
	Human	0.24	0.22	0.27
Prison	Both	0.29	0.27	0.31
	Alg.	0.08	0.07	0.09
	Human	0.38	0.36	0.40
Unempl.	Both	0.41	0.39	0.43
	Alg.	0.25	0.23	0.27

(c) Outcome category "A little fair"

Context	Decider	$\hat{P}(y=2)$	95% CI	
	Human	0.41	0.40	0.43
Bank	Both	0.36	0.34	0.38
	Alg.	0.44	0.43	0.45
	Human	0.44	0.43	0.45
Job	Both	0.44	0.43	0.45
	Alg.	0.38	0.36	0.40
	Human	0.44	0.43	0.45
Prison	Both	0.44	0.43	0.45
	Alg.	0.34	0.32	0.36
	Human	0.40	0.38	0.41
Unempl.	Both	0.37	0.36	0.39
	Alg.	0.44	0.43	0.45

(d) Outcome category "Not at all fair"

Context	Decider	$\hat{P}(y=1)$	95% CI	
	Human	0.16	0.14	0.18
Bank	Both	0.10	0.08	0.11
	Alg.	0.24	0.22	0.27
	Human	0.28	0.26	0.31
Job	Both	0.26	0.24	0.29
	Alg.	0.51	0.48	0.54
	Human	0.28	0.26	0.31
Prison	Both	0.23	0.21	0.26
	Alg.	0.57	0.54	0.60
	Human	0.14	0.12	0.16
Unempl.	Both	0.11	0.10	0.13
	Alg.	0.28	0.25	0.30

B. Supplemental Experimental Procedures

B.1. Vignette Bank

B.1.1. Decision-maker: Algorithm; Data: Internet data; Action: Punitive

German Prompt No.1: Eine Bank hat ein Computerprogramm zur Vergabe von Krediten an Privatpersonen entwickelt. Dieses Programm verwendet Daten über vergangene Kredite der Person sowie im Internet verfügbare Informationen über die Person. Das Programm vergleicht diese Informationen mit denen von anderen Personen, die bereits Kredite erhalten haben. Das Programm entscheidet automatisch, ob die Person einen beantragten Kredit erhält.

English Translation Prompt No.1: *A bank developed a computer-program to assist the distribution of credits to private citizens. This program uses data about the credit history and data available on the internet about the person. This program compares the information with the that of other people that already received loans in the past. The program determines automatically whether the person receives the requested credit.*

B.1.2. Decision-maker: Both; Data: Internet data; Action: Punitive

German Prompt No.2: Eine Bank hat ein Computerprogramm zur Vergabe von Krediten an Privatpersonen entwickelt. Dieses Programm verwendet Daten über vergangene Kredite der Person sowie im Internet verfügbare Informationen über die Person. Das Programm vergleicht diese Informationen mit denen von anderen Personen, die bereits Kredite erhalten haben. Das Programm schlägt einem Mitarbeiter vor, ob die Person einen beantragten Kredit erhalten soll. Die endgültige Entscheidung wird vom Mitarbeiter getroffen.

English Translation Prompt No.2: *A bank developed a computer-program to assist the distribution of credits to private citizens. This program uses data about the credit history and data available on the internet about the person. This program compares the information with the that of other people that already received loans in the past. The program gives an employee a recommendation whether the person should receive the loan or not. The employee makes the final decision.*

B.1.3. Decision-maker: Human; Data: Internet data; Action: Punitive

German Prompt No.3: Eine Bank hat ein Computerprogramm zur Vergabe von Krediten an Privatpersonen entwickelt. Dieses Programm verwendet Daten über vergangene Kredite der Person sowie im Internet verfügbare Informationen über die Person. Ein Mitarbeiter der Bank vergleicht diese Informationen mit denen von anderen Personen, die bereits Kredite erhalten haben. Der Mitarbeiter entscheidet, ob die Person einen beantragten Kredit erhält.

English Translation Prompt No.3: *A bank developed a computer-program to assist the distribution of credits to private citizens. This program uses data about the credit history and data available on the internet about the person. An employee compares the information with the that of other people that already received loans in the past. The employee determines whether the person receives the requested credit.*

B.1.4. Decision-maker: Algorithm; Data: No Internet data; Action: Punitive

German Prompt No.4: Eine Bank hat ein Computerprogramm zur Vergabe von Krediten an Privatpersonen entwickelt. Dieses Programm verwendet Daten über vergangene Kredite der Person. Das Programm vergleicht diese Informationen mit denen von anderen Personen, die bereits Kredite erhalten haben. Das Programm entscheidet automatisch, ob die Person einen beantragten Kredit erhält.

English Translation Prompt No.4: *A bank developed a computer-program to assist the distribution of credits to private citizens. This program uses data about the credit history about the person. The program compares the information with the that of other people that already received loans in the past. The program determines automatically whether the person receives the requested credit.*

B.1.5. Decision-maker: Both; Data: No Internet data; Action: Punitive

German Prompt No.5: Eine Bank hat ein Computerprogramm zur Vergabe von Krediten an Privatpersonen entwickelt. Dieses Programm verwendet Daten über vergangene Kredite der Person. Das Programm vergleicht diese Informationen mit denen von anderen Personen, die bereits Kredite erhalten haben. Das Programm schlägt einem Mitarbeiter vor, ob die Person einen beantragten Kredit erhalten soll. Die endgültige Entscheidung wird vom Mitarbeiter getroffen.

English Translation Prompt No.5: *A bank developed a computer-program to assist the distribution of credits to private citizens. This program uses data about the credit history about the person. The program compares the information with the that of other people that already received loans in the past. The program gives an employee a recommendation whether the person should receive the loan or not. The employee determines whether the person receives the requested credit.*

B.1.6. Decision-maker: Human; Data: No Internet data; Action: Punitive

German Prompt No.6: Eine Bank hat ein Computerprogramm zur Vergabe von Krediten an Privatpersonen entwickelt. Dieses Programm verwendet Daten über vergangene Kredite der Person. Ein Mitarbeiter der Bank vergleicht diese Informationen mit denen von anderen Personen, die bereits Kredite erhalten haben. Der Mitarbeiter entscheidet, ob die Person einen beantragten Kredit erhält.

English Translation Prompt No.6: *A bank developed a computer-program to assist the distribution of credits to private citizens. This program uses data about the credit history about the person. The employee compares the information with the that of other people that already received loans in the past. The employee determines whether the person receives the requested credit.*

B.1.7. Decision-maker: Algorithm; Data: No Internet data; Action: Assistive

German Prompt No.7: Eine Bank hat ein Computerprogramm zum Angebot von exklusiven Finanzprodukten an Privatpersonen entwickelt. Dieses Programm verwendet Daten über frühere Finanzentscheidungen der Person sowie im Internet verfügbare Informationen über die Person. Das Programm vergleicht diese Informationen mit denen von anderen Personen, die ähnliche Finanzprodukte nutzen. Das Programm entscheidet automatisch, ob der Person das exklusive Finanzprodukt angeboten wird.

English Translation Prompt No.7: *A bank developed a computer-program to offer exclusive financial products to private citizens. This program uses data about the credit history and data available on the internet about the person. The program compares the information with the that of people who use similar financial products in the past. The program determines whether the person receives the exclusive financial product offer.*

B.1.8. Decision-maker: Both; Data: Internet data; Action: Assistive

German Prompt No.8: Eine Bank hat ein Computerprogramm zum Angebot von exklusiven Finanzprodukten an Privatpersonen entwickelt. Dieses Programm verwendet Daten über frühere Finanzentscheidungen der Person sowie im Internet verfügbare Informationen über die Person. Das Programm vergleicht diese Informationen mit denen von anderen Personen, die ähnliche Finanzprodukte nutzen. Das Programm schlägt einem Mitarbeiter vor, ob der Person das exklusive Finanzprodukt angeboten werden soll. Die endgültige Entscheidung wird vom Mitarbeiter getroffen.

English Translation Prompt No.8: *A bank developed a computer-program to offer exclusive financial products to private citizens. This program uses data about the credit history data available on the internet about the person. The program compares the information with the that of other people that use similar financial products. The program gives an employee a recommendation whether the person should receive the exclusive financial product offer. The employee determines the final decision.*

B.1.9. Decision-maker: Human; Data: Internet data; Action: Assistive

German Prompt No.9: Eine Bank hat ein Computerprogramm zum Angebot von exklusiven Finanzprodukten an Privatpersonen entwickelt. Dieses Programm verwendet Daten über frühere Finanzentscheidungen der Person sowie im Internet verfügbare Informationen über die Person. Ein Mitarbeiter der Bank vergleicht diese Informationen

mit denen von anderen Personen, die ähnliche Finanzprodukte nutzen. Der Mitarbeiter entscheidet, ob der Person das exklusive Finanzprodukt angeboten wird.

English Translation Prompt No.9: *A bank developed a computer-program to offer exclusive financial products to private citizens. This program uses data about the credit history and data available on the internet about the person. The employee compares the information with the that of other people that use similar financial products. The employee determines whether the person receives the exclusive financial product offer.*

B.1.10. Decision-maker: Algorithm; Data: No Internet data; Action: Assistive

German Prompt No.10: Eine Bank hat ein Computerprogramm zum Angebot von exklusiven Finanzprodukten an Privatpersonen entwickelt. Dieses Programm verwendet Daten über frühere Finanzentscheidungen der Person. Das Programm vergleicht diese Informationen mit denen von anderen Personen, die ähnliche Finanzprodukte nutzen. Das Programm entscheidet automatisch, ob der Person das exklusive Finanzprodukt angeboten wird.

English Translation Prompt No.10: *A bank developed a computer-program to offer exclusive financial products to private citizens. This program uses data about the credit history about the person. The program compares the information with the that of other people that use similar financial products. The program gives an employee a recommendation whether the person should receive the loan or not. The program determines whether the person receives the exclusive financial product offer.*

B.1.11. Decision-maker: Both; Data: No Internet data; Action: Assistive

German Prompt No.11: Eine Bank hat ein Computerprogramm zum Angebot von exklusiven Finanzprodukten an Privatpersonen entwickelt. Dieses Programm verwendet Daten über frühere Finanzentscheidungen der Person. Das Programm vergleicht diese Informationen mit denen von anderen Personen, die ähnliche Finanzprodukte nutzen. Das Programm schlägt einem Mitarbeiter vor, ob der Person das exklusive Finanzprodukt angeboten werden soll. Die endgültige Entscheidung wird vom Mitarbeiter getroffen.

English Translation Prompt No.11: *A bank developed a computer-program to offer exclusive financial products to private citizens. This program uses data about the prior financial decisions. The program compares the information with the that of other people that use similar financial products. The program gives an employee a recommendation whether the person should receive the exclusive financial product offer. The employee determines the final decision.*

B.1.12. Decision-maker: Human; Data: No Internet data; Action: Assistive

German Prompt No.12: Eine Bank hat ein Computerprogramm zum Angebot von exklusiven Finanzprodukten an Privatpersonen entwickelt. Dieses Programm verwendet Daten über frühere Finanzentscheidungen der Person. Ein Mitarbeiter der Bank vergleicht diese Informationen mit denen von anderen Personen, die ähnliche Finanzprodukte nutzen. Der Mitarbeiter entscheidet, ob der Person das exklusive Finanzprodukt angeboten wird.

English Translation Prompt No.12: *A bank developed a computer-program to offer exclusive financial products to private citizens. This program uses data about the credit history about the person. The employee compares the information with the that of other people that use similar financial products. The employee determines whether the person receives the exclusive financial product offer.*

B.2. Vignette Prison

B.2.1. Decision-maker: Algorithm; Data: Internet data; Action: Assistive

German Prompt No. 1: Eine Behörde hat ein Computerprogramm zur Entscheidung über die vorzeitige Entlassung von Strafgefangenen entwickelt. Dieses Programm verwendet Daten über den Lebenslauf der Person sowie im Internet verfügbare Informationen über die Person. Das Programm vergleicht diese Informationen mit denen von anderen Personen, die bereits frühzeitig entlassen wurden. Das Programm entscheidet automatisch, ob die Person vorzeitig aus der Haft entlassen wird.

English Translation Prompt No.1: *A government agency has developed a program for the early release of inmates. The program uses data about the life of a person and publicly available information from the internet. The program compares this information with that of other people that already got an early release. The program determines automatically, whether the person gets an early release.*

B.2.2. Decision-maker: Both; Data: Internet data; Action: Assistive

German Prompt No. 2: Eine Behörde hat ein Computerprogramm zur Entscheidung über die vorzeitige Entlassung von Strafgefangenen entwickelt. Dieses Programm verwendet Daten über den Lebenslauf der Person sowie im Internet verfügbare Informationen über die Person. Das Programm vergleicht diese Informationen mit denen von anderen Personen, die bereits frühzeitig entlassen wurden. Das Programm schlägt einem Beamten vor, ob die Person vorzeitig aus der Haft entlassen werden sollte. Die endgültige Entscheidung wird vom Richter getroffen.

English Translation Prompt No.2: *A government agency has developed a program for the early release of inmates. The program uses data about the life of a person and publicly available information from the internet. The program compares this information*

with that of other people that already got an early release. The program gives a government worker a recommendation on whether the person should get an early release. The final decision is made by the judge.

B.2.3. Decision-maker: Human; Data: Internet data; Action: Assistive

German Prompt No. 3: Eine Behörde hat ein Computerprogramm zur Entscheidung über die vorzeitige Entlassung von Strafgefangenen entwickelt. Dieses Programm verwendet Daten über den Lebenslauf der Person sowie im Internet verfügbare Informationen über die Person. Ein Beamter vergleicht diese Informationen mit denen von anderen Personen, die bereits frühzeitig entlassen wurden. Der Richter entscheidet, ob die Person vorzeitig aus der Haft entlassen wird.

English Translation Prompt No.3: *A government agency has developed a program for the early release of inmates. The program uses data about the life of a person and publicly available information from the internet. A government worker compares this information with that of other people that already got an early release. The judge determines whether the person gets an early release.*

B.2.4. Decision-maker: Algorithm; Data: No Internet data; Action: Assistive

German Prompt No. 4: Eine Behörde hat ein Computerprogramm zur Entscheidung über die vorzeitige Entlassung von Strafgefangenen entwickelt. Dieses Programm verwendet Daten über den Lebenslauf der Person. Das Programm vergleicht diese Informationen mit denen von anderen Personen, die bereits frühzeitig entlassen wurden. Das Programm entscheidet automatisch, ob die Person vorzeitig aus der Haft entlassen wird.

English Translation Prompt No.4: *A government agency has developed a program for the early release of inmates. The program uses data about the life of a person. The program compares this information with that of other people that already got an early release. The program determines automatically, whether the person gets an early release.*

B.2.5. Decision-maker: Both; Data: No Internet data; Action: Assistive

German Prompt No. 5: Eine Behörde hat ein Computerprogramm zur Entscheidung über die vorzeitige Entlassung von Strafgefangenen entwickelt. Dieses Programm verwendet Daten über den Lebenslauf der Person. Das Programm vergleicht diese Informationen mit denen von anderen Personen, die bereits frühzeitig entlassen wurden. Das Programm schlägt einem Beamten vor, ob die Person vorzeitig aus der Haft entlassen werden sollte. Die endgültige Entscheidung wird vom Richter getroffen.

English Translation Prompt No.5: *A government agency has developed a program for the early release of inmates. The program uses data about the life of a person. The*

program compares this information with that of other people that already got an early release. The program gives a government worker a recommendation on whether the person should get an early release. The final decision is made by the judge.

B.2.6. Decision-maker: Human; Data: No Internet data; Action: Assistive

German Prompt No. 6: Eine Behörde hat ein Computerprogramm zur Entscheidung über die vorzeitige Entlassung von Strafgefangenen entwickelt. Dieses Programm verwendet Daten über den Lebenslauf der Person. Ein Beamter vergleicht diese Informationen mit denen von anderen Personen, die bereits frühzeitig entlassen wurden. Der Richter entscheidet, ob die Person vorzeitig aus der Haft entlassen wird.

English Translation Prompt No.6: *A government agency has developed a program for the early release of inmates. The program uses data about the life of a person. A government worker compares this information with that of other people that already got an early release. The judge determines whether the person gets an early release.*

B.3. Vignette Job

B.3.1. Decision-maker: Algorithm; Data: Internet data; Action: Assistive

German Prompt No.1: Ein Unternehmen hat ein Computerprogramm zur Entscheidung über die Einstellung von neuen Mitarbeitern entwickelt. Dieses Programm verwendet Daten über den Lebenslauf der Person sowie im Internet verfügbare Informationen über die Person. Das Programm vergleicht diese Informationen mit denen von anderen Personen, die bereits im Unternehmen tätig sind. Das Programm entscheidet automatisch, ob die Person eingestellt wird.

English Translation Prompt No.1: *A company has developed a computer program to hire new employees. The program uses the vitae and publicly available information from the internet of people. The program compares the information with that of people that are already working in the company. The program determines automatically whether the person will get hired.*

B.3.2. Decision-maker: Both; Data: Internet data; Action: Assistive

German Prompt No.2: Ein Unternehmen hat ein Computerprogramm zur Entscheidung über die Einstellung von neuen Mitarbeitern entwickelt. Dieses Programm verwendet Daten über den Lebenslauf der Person sowie im Internet verfügbare Informationen über die Person. Das Programm vergleicht diese Informationen mit denen von anderen Personen, die bereits im Unternehmen tätig sind. Das Programm schlägt einem Mitarbeiter der Personalabteilung vor, ob die Person eingestellt werden sollte. Die endgültige Entscheidung wird vom Mitarbeiter getroffen.

English Translation Prompt No.2: *A company has developed a computer program*

to hire new employees. The program uses the vitae and publicly available information from the internet of people. The program compares the information with that of people that are already working in the company. The program gives a recommendation to an employee of HR whether to hire the candidate. An employee determines whether the person will get hired.

B.3.3. Decision-maker: Human; Data: Internet data; Action: Assistive

German Prompt No.3: Ein Unternehmen hat ein Computerprogramm zur Entscheidung über die Einstellung von neuen Mitarbeitern entwickelt. Dieses Programm verwendet Daten über den Lebenslauf der Person sowie im Internet verfügbare Informationen über die Person. Ein Mitarbeiter der Personalabteilung vergleicht diese Informationen mit denen von anderen Personen, die bereits im Unternehmen tätig sind. Der Mitarbeiter entscheidet, ob die Person eingestellt wird.

English Translation Prompt No.3: *A company has developed a computer program to hire new employees. The program uses the vitae and publicly available information from the internet of people. The employee compares the information with that of people that are already working in the company. An employee determines whether the person will get hired.*

B.3.4. Decision-maker: Algorithm; Data: No Internet data; Action: Assistive

German Prompt No.4: Ein Unternehmen hat ein Computerprogramm zur Entscheidung über die Einstellung von neuen Mitarbeitern entwickelt. Dieses Programm verwendet Daten über den Lebenslauf der Person. Das Programm vergleicht diese Informationen mit denen von anderen Personen, die bereits im Unternehmen tätig sind. Das Programm entscheidet automatisch, ob die Person eingestellt wird.

English Translation Prompt No.4: *A company has developed a computer program to hire new employees. The program uses the vitae of people. The program compares the information with that of people that are already working in the company. The program determines automatically whether the person will get hired.*

B.3.5. Decision-maker: Both; Data: No Internet data; Action: Assistive

German Prompt No.5: Ein Unternehmen hat ein Computerprogramm zur Entscheidung über die Einstellung von neuen Mitarbeitern entwickelt. Dieses Programm verwendet Daten über den Lebenslauf der Person. Das Programm vergleicht diese Informationen mit denen von anderen Personen, die bereits im Unternehmen tätig sind. Das Programm schlägt einem Mitarbeiter der Personalabteilung vor, ob die Person eingestellt werden sollte. Die endgültige Entscheidung wird vom Mitarbeiter getroffen.

English Translation Prompt No.5: *A company has developed a computer program to hire new employees. The program uses the vitae of people. The program compares the information with that of people that are already working in the company. The program gives an recommendation to an employee. The employee determines whether the person will get hired.*

B.3.6. Decision-maker: Human; Data: No Internet data; Action: Assistive

German Prompt No.6: Ein Unternehmen hat ein Computerprogramm zur Entscheidung über die Einstellung von neuen Mitarbeitern entwickelt. Dieses Programm verwendet Daten über den Lebenslauf der Person. Ein Mitarbeiter der Personalabteilung vergleicht diese Informationen mit denen von anderen Personen, die bereits im Unternehmen tätig sind. Der Mitarbeiter entscheidet, ob die Person eingestellt wird.

English Translation Prompt No.6: *A company has developed a computer program to hire new employees. The program uses the vitae of people. An employee of HR compares the information with that of people that are already working in the company. The employee determines whether the person will get hired.*

B.3.7. Decision-maker: Algorithm; Data: Internet data; Action: Punitive

German Prompt No.7: Ein Unternehmen hat ein Computerprogramm zur Entscheidung über die Entlassung von Mitarbeitern in ihrer Probezeit entwickelt. Dieses Programm verwendet Daten über die bisherige Leistung der Person am Arbeitsplatz sowie im Internet verfügbare Informationen über die Person. Das Programm vergleicht diese Informationen mit denen von anderen Personen, die im Unternehmen tätig sind. Das Programm entscheidet automatisch, ob die Person entlassen wird.

English Translation Prompt No.7: *A company has developed a computer program to fire employees in their trial-period. The program uses data from the prior performance of an employee at the company and public information from the internet about the person. A program compares the information with that of people that are already working in the company. The program determines automatically whether the person will get laid off.*

B.3.8. Decision-maker: Both; Data: Internet data; Action: Punitive

German Prompt No.8: Ein Unternehmen hat ein Computerprogramm zur Entscheidung über die Entlassung von Mitarbeitern in ihrer Probezeit entwickelt. Dieses Programm verwendet Daten über die bisherige Leistung der Person am Arbeitsplatz sowie im Internet verfügbare Informationen über die Person. Das Programm vergleicht diese Informationen mit denen von anderen Personen, die im Unternehmen tätig sind. Das Programm schlägt einem Mitarbeiter der Personalabteilung vor, ob die Person entlassen werden sollte. Die endgültige Entscheidung wird vom Mitarbeiter getroffen.

English Translation Prompt No.8: *A company has developed a computer program to fire employees in their trial-period. The program uses data from the prior performance of an employee at the company and public information from the internet about the person. A program compares the information with that of people that are already working in the company. The program gives an employee of HR a recommendation. The employee determines whether the person will get laid off.*

B.3.9. Decision-maker: Human; Data: Internet data; Action: Punitive

German Prompt No.9: Ein Unternehmen hat ein Computerprogramm zur Entscheidung über die Entlassung von Mitarbeitern in ihrer Probezeit entwickelt. Dieses Programm verwendet Daten über die bisherige Leistung der Person am Arbeitsplatz sowie im Internet verfügbare Informationen über die Person. Ein Mitarbeiter der Personalabteilung vergleicht diese Informationen mit denen von anderen Personen, die im Unternehmen tätig sind. Der Mitarbeiter entscheidet, ob die Person entlassen wird.

English Translation Prompt No.9: *A company has developed a computer program to fire employees in their trial-period. The program uses data from the prior performance of an employee at the company and public information from the internet about the person. An employee of HR compares the information with that of people that are already working in the company. The program gives an employee of HR a recommendation. The employee determines whether the person will get laid off.*

B.3.10. Decision-maker: Algorithm; Data: No Internet data; Action: Punitive

German Prompt No.10: Ein Unternehmen hat ein Computerprogramm zur Entscheidung über die Entlassung von Mitarbeitern in ihrer Probezeit entwickelt. Dieses Programm verwendet Daten über die bisherige Leistung der Person am Arbeitsplatz. Das Programm vergleicht diese Informationen mit denen von anderen Personen, die im Unternehmen tätig sind. Das Programm entscheidet automatisch, ob die Person entlassen wird.

English Translation Prompt No.10: *A company has developed a computer program to fire employees in their trial-period. The program uses data from the prior performance of an employee at the company. The program compares the information with that of people that are already working in the company. The program determines automatically whether the person will get laid off.*

B.3.11. Decision-maker: Both; Data: No Internet data; Action: Punitive

German Prompt No.11: Ein Unternehmen hat ein Computerprogramm zur Entscheidung über die Entlassung von Mitarbeitern in ihrer Probezeit entwickelt. Dieses Programm verwendet Daten über die bisherige Leistung der Person am Arbeitsplatz. Das

Programm vergleicht diese Informationen mit denen von anderen Personen, die im Unternehmen tätig sind. Das Programm schlägt einem Mitarbeiter der Personalabteilung vor, ob die Person entlassen werden sollte. Die endgültige Entscheidung wird vom Mitarbeiter getroffen.

English Translation Prompt No.11: *A company has developed a computer program to fire employees in their trial-period. The program uses data from the prior performance of an employee at the company. The program compares the information with that of people that are already working in the company. The program gives an employee of HR a recommendation of whether to fire the person. The employee determines whether the person will get laid off.*

B.3.12. Decision-maker: Human; Data: No Internet data; Action: Punitive

German Prompt No.12: Ein Unternehmen hat ein Computerprogramm zur Entscheidung über die Entlassung von Mitarbeitern in ihrer Probezeit entwickelt. Dieses Programm verwendet Daten über die bisherige Leistung der Person am Arbeitsplatz. Ein Mitarbeiter der Personalabteilung vergleicht diese Informationen mit denen von anderen Personen, die im Unternehmen tätig sind. Der Mitarbeiter entscheidet, ob die Person entlassen wird.

English Translation Prompt No.12: *A company has developed a computer program to fire employees in their trial-period. The program uses data from the prior performance of an employee at the company. An employee of HR compares the information with that of people that are already working in the company. The employee determines whether the person will get laid off.*

B.4. Vignette Unemployed

B.4.1. Decision-maker: Algorithm; Data: Internet data; Action: Assistive

German Prompt No.1: Eine lokale Arbeitsagentur hat ein Computerprogramm zur Vergabe von Unterstützungsmaßnahmen von Arbeitssuchenden entwickelt. Dieses Programm verwendet Daten über vergangene Phasen der Arbeitstätigkeit und Arbeitslosigkeit der Person sowie im Internet verfügbare Informationen über die Person. Das Programm vergleicht diese Informationen mit denen von anderen arbeitssuchenden Personen, die erfolgreich an einer Maßnahme teilgenommen haben. Das Programm entscheidet automatisch, ob die Person eine Unterstützungsmaßnahme erhält

English Translation Prompt No.1: *A local employment agency has developed a computer program for assigning support measures to job seekers. The program uses data about the work and unemployment history of the person and publicly available information from the internet. The program compares this information with that of other unemployed and labour seeking seeking people that have successfully undertaken a measure. The program determines automatically whether the person will get support measures.*

B.4.2. Decision-maker: Human; Data: Internet data; Action: Assistive

German Prompt No.2: Eine lokale Arbeitsagentur hat ein Computerprogramm zur Vergabe von Unterstützungsmaßnahmen von Arbeitssuchenden entwickelt. Dieses Programm verwendet Daten über vergangene Phasen der Arbeitstätigkeit und Arbeitslosigkeit der Person sowie im Internet verfügbare Informationen über die Person. Ein Mitarbeiter der Arbeitsagentur vergleicht diese Informationen mit denen von anderen arbeitssuchenden Personen, die erfolgreich an einer Maßnahme teilgenommen haben. Der Mitarbeiter entscheidet, ob die Person eine Unterstützungsmaßnahme erhält

English Translation Prompt No.2: *A local employment agency has developed a computer program for assigning support measures to job seekers. The program uses data about the work and unemployment history of the person and publicly available information from the internet. An employee of the unemployment agency compares this information with that of other unemployed and labour seeking seeking people that have successfully undertaken a measure. The employee determines whether the person will get support measures.*

B.4.3. Decision-maker: Both; Data: Internet data; Action: Assistive

German Prompt No.3: Eine lokale Arbeitsagentur hat ein Computerprogramm zur Vergabe von Unterstützungsmaßnahmen von Arbeitssuchenden entwickelt. Dieses Programm verwendet Daten über vergangene Phasen der Arbeitstätigkeit und Arbeitslosigkeit der Person sowie im Internet verfügbare Informationen über die Person. Das Programm vergleicht diese Informationen mit denen von anderen arbeitssuchenden Personen, die erfolgreich an einer Maßnahme teilgenommen haben. Das Programm schlägt einem Mitarbeiter der Arbeitsagentur vor, ob die Person eine Unterstützungsmaßnahme erhält. Die endgültige Entscheidung wird vom Mitarbeiter getroffen.

English Translation Prompt No.3: *A local employment agency has developed a computer program for assigning support measures to job seekers. The program uses data about the work and unemployment history of the person and publicly available information from the internet. The program compares this information with that of other unemployed and labour seeking seeking people that have successfully undertaken a measure. The program gives an employee of the unemployment agency a recommendation on whether a person gets labour assistance or not. The employee makes the final decision whether the person will get support measures.*

B.4.4. Decision-maker: Algorithm; Data: No Internet data; Action: Assistive

German Prompt No.4: Eine lokale Arbeitsagentur hat ein Computerprogramm zur Vergabe von Unterstützungsmaßnahmen von Arbeitssuchenden entwickelt. Dieses Programm verwendet Daten über vergangene Phasen der Arbeitstätigkeit und Arbeitslosigkeit

der Person. Das Programm vergleicht diese Informationen mit denen von anderen arbeitssuchenden Personen, die erfolgreich an einer Maßnahme teilgenommen haben. Das Programm entscheidet automatisch, ob die Person eine Unterstützungsmaßnahme erhält.

English Translation Prompt No.4: *A local employment agency has developed a computer program for assigning support measures to job seekers. The program uses data about the work and unemployment history of the person. The program compares this information with that of other unemployed and labour seeking seeking people that have successfully undertaken a measure. The program determines automatically whether the person will get support measures.*

B.4.5. Decision-maker: Human; Data: No Internet data; Action: Assistive

German Prompt No.5: Eine lokale Arbeitsagentur hat ein Computerprogramm zur Vergabe von Unterstützungsmaßnahmen von Arbeitssuchenden entwickelt. Dieses Programm verwendet Daten über vergangene Phasen der Arbeitstätigkeit und Arbeitslosigkeit der Person. Ein Mitarbeiter der Arbeitsagentur vergleicht diese Informationen mit denen von anderen arbeitssuchenden Personen, die erfolgreich an einer Maßnahme teilgenommen haben. Der Mitarbeiter entscheidet, ob die Person eine Unterstützungsmaßnahme erhält.

English Translation Prompt No.5: *A local employment agency has developed a computer program for assigning support measures to job seekers. The program uses data about the work and unemployment history of the person. The program compares this information with that of other unemployed and labour seeking seeking people that have successfully undertaken a measure. The program determines automatically whether the person will get support measures.*

B.4.6. Decision-maker: Both; Data: No Internet data; Action: Assistive

German Prompt No.6: Eine lokale Arbeitsagentur hat ein Computerprogramm zur Vergabe von Unterstützungsmaßnahmen von Arbeitssuchenden entwickelt. Dieses Programm verwendet Daten über vergangene Phasen der Arbeitstätigkeit und Arbeitslosigkeit der Person. Das Programm vergleicht diese Informationen mit denen von anderen arbeitssuchenden Personen, die erfolgreich an einer Maßnahme teilgenommen haben. Das Programm schlägt einem Mitarbeiter der Arbeitsagentur vor, ob die Person eine Unterstützungsmaßnahme erhält. Die endgültige Entscheidung wird vom Mitarbeiter getroffen.

English Translation Prompt No.6: *A local employment agency has developed a computer program for assigning support measures to job seekers. The program uses data about the work and unemployment history of the person. The program compares this information with that of other unemployed and labour seeking seeking people that have*

successfully undertaken a measure. The program gives an employee of the unemployment agency a recommendation on whether a person gets labour assistance or not. The employee makes the final decision whether the person will get support measures.

B.4.7. Decision-maker: Algorithm; Data: Internet data; Action: Punitive

German Prompt No.7: Eine lokale Arbeitsagentur hat ein Computerprogramm zur Entscheidung der Kürzung von Arbeitslosengeld in Folge von Verletzungen von Mitwirkungs- und Verhaltenspflichten einer arbeitslosen Person entwickelt. Dieses Programm verwendet Daten über das Verhalten der Person bei der Arbeitssuche und Maßnahmenteilnahme sowie im Internet verfügbare Informationen über die Person. Das Programm vergleicht diese Informationen mit denen von anderen arbeitslosen Personen. Das Programm entscheidet automatisch, ob der Person das Arbeitslosengeld gekürzt wird.

English Translation Prompt No.7: *A local unemployment agency has developed a computer program to determine the cut of unemployment benefits for injuries given contributions and behavioural obligations of unemployed people. The program uses data about the work search behaviour and the participation history of the person and publicly available information from the internet. The program compares this information with that of other unemployed people. The program determines automatically whether the person will loss part of the unemployment benefits.*

B.4.8. Decision-maker: Both; Data: Internet data; Action: Punitive

German Prompt No.8: Eine lokale Arbeitsagentur hat ein Computerprogramm zur Entscheidung der Kürzung von Arbeitslosengeld in Folge von Verletzungen von Mitwirkungs- und Verhaltenspflichten einer arbeitslosen Person entwickelt. Dieses Programm verwendet Daten über das Verhalten der Person bei der Arbeitssuche und Maßnahmenteilnahme sowie im Internet verfügbare Informationen über die Person. Das Programm vergleicht diese Informationen mit denen von anderen arbeitslosen Personen. Das Programm schlägt einem Mitarbeiter der Arbeitsagentur vor, ob der Person das Arbeitslosengeld gekürzt werden soll. Die endgültige Entscheidung wird vom Mitarbeiter getroffen.

English Translation Prompt No.8: *A local unemployment agency has developed a computer program to determine the cut of unemployment benefits for injuries given contributions and behavioural obligations of unemployed people. The program uses data about the work search behaviour and the participation history of the person and publicly available information from the internet. The program compares this information with that of other unemployed people. The program gives an employee of the unemployment agency a recommendation on whether a person gets less unemployment benefits or not. An employee determines whether the person will loss part of the unemployment benefits.*

B.4.9. Decision-maker: Human; Data: Internet data; Action: Punitive

German Prompt No.9: Eine lokale Arbeitsagentur hat ein Computerprogramm zur Entscheidung der Kürzung von Arbeitslosengeld in Folge von Verletzungen von Mitwirkungs- und Verhaltenspflichten einer arbeitslosen Person entwickelt. Dieses Programm verwendet Daten über das Verhalten der Person bei der Arbeitssuche und Maßnahmenteilnahme sowie im Internet verfügbare Informationen über die Person. Ein Mitarbeiter der Arbeitsagentur vergleicht diese Informationen mit denen von anderen arbeitslosen Personen. Der Mitarbeiter entscheidet, ob der Person das Arbeitslosengeld gekürzt wird.

English Translation Prompt No.9: *A local unemployment agency has developed a computer program to determine the cut of unemployment benefits for injuries given contributions and behavioural obligations of unemployed people. The program uses data about the work search behaviour and the participation history of the person and publicly available information from the internet. An employee of the unemployment agency compares this information with that of other unemployed people. An employee determines whether the person will loss part of the unemployment benefits.*

B.4.10. Decision-maker: Algorithm; Data: No Internet data; Action: Punitive

German Prompt No.10: Eine lokale Arbeitsagentur hat ein Computerprogramm zur Entscheidung der Kürzung von Arbeitslosengeld in Folge von Verletzungen von Mitwirkungs- und Verhaltenspflichten einer arbeitslosen Person entwickelt. Dieses Programm verwendet Daten über das Verhalten der Person bei der Arbeitssuche und Maßnahmenteilnahme. Das Programm vergleicht diese Informationen mit denen von arbeitslosen Personen. Das Programm entscheidet automatisch, ob der Person das Arbeitslosengeld gekürzt wird.

English Translation Prompt No.10: *A local unemployment agency has developed a computer program to determine the cut of unemployment benefits for injuries given contributions and behavioural obligations of unemployed people. The program uses data about the work search behaviour and the participation history of the person. An employee of the unemployment agency compares this information with that of other unemployed people. The program determines automatically whether the person will loss part of the unemployment benefits.*

B.4.11. Decision-maker: Both; Data: No Internet data; Action: Punitive

German Prompt No.11: Eine lokale Arbeitsagentur hat ein Computerprogramm zur Entscheidung der Kürzung von Arbeitslosengeld in Folge von Verletzungen von Mitwirkungs- und Verhaltenspflichten einer arbeitslosen Person entwickelt. Dieses Programm verwendet Daten über das Verhalten der Person bei der Arbeitssuche und Maßnahmenteilnahme. Das Programm vergleicht diese Informationen mit denen von anderen arbeitslosen Personen. Das Programm schlägt einem Mitarbeiter der Arbeitsagentur vor,

ob der Person das Arbeitslosengeld gekürzt werden soll. Die endgültige Entscheidung wird vom Mitarbeiter getroffen.

English Translation Prompt No.11: *A local unemployment agency has developed a computer program to determine the cut of unemployment benefits for injuries given contributions and behavioural obligations of unemployed people. The program uses data about the work search behaviour and the participation history of the person. The program compares this information with that of other unemployed people. The program gives an employee of the unemployment agency a recommendation on whether a person gets less unemployment benefits or not. An employee determines whether the person will loss part of the unemployment benefits.*

B.4.12. Decision-maker: Human; Data: No Internet data; Action: Punitive

German Prompt No.12: Eine lokale Arbeitsagentur hat ein Computerprogramm zur Entscheidung der Kürzung von Arbeitslosengeld in Folge von Verletzungen von Mitwirkungs- und Verhaltenspflichten einer arbeitslosen Person entwickelt. Dieses Programm verwendet Daten über das Verhalten der Person bei der Arbeitssuche und Maßnahmenteilnahme. Ein Mitarbeiter der Arbeitsagentur vergleicht diese Informationen mit denen von anderen arbeitslosen Personen. Der Mitarbeiter entscheidet, ob der Person das Arbeitslosengeld gekürzt wird.

English Translation Prompt No.12: *A local unemployment agency has developed a computer program to determine the cut of unemployment benefits for injuries given contributions and behavioural obligations of unemployed people. The program uses data about the work search behaviour and the participation history of the person. An employee of the unemployment agency compares this information with that of other unemployed people. An employee determines whether the person will loss part of the unemployment benefits.*
